# A Pain eHealth Platform for Engaging Obese, Older Adults with Chronic Low Back Pain in Nonpharmacological Pain Treatments: Protocol for a Pilot Feasibility Study

**DOI:** 10.2196/14525

**Published:** 2020-01-02

**Authors:** Amber K Brooks, David P Miller Jr, Jason T Fanning, Erin L Suftin, M Carrington Reid, Brian J Wells, Xiaoyan Leng, Robert W Hurley

**Affiliations:** 1 Department of Anesthesiology Wake Forest School of Medicine Winston Salem, NC United States; 2 Department of General Internal Medicine Wake Forest School of Medicine Winston Salem, NC United States; 3 Department of Health and Exercise Science Wake Forest University Winston Salem, NC United States; 4 Department of Internal Medicine Section on Gerontology and Geriatric Medicine Wake Forest School of Medicine Winston Salem, NC United States; 5 Division of Public Health Sciences Wake Forest School of Medicine Winston Salem, NC United States; 6 Division of Geriatric and Palliative Medicine Weill Cornell Medicine New York, NY United States

**Keywords:** chronic pain, patient portal, Web-based treatments, electronic health records

## Abstract

**Background:**

Low back pain is a costly healthcare problem and the leading cause of disability among adults in the United States. Primary care providers urgently need effective ways to deliver evidence-based, nonpharmacological therapies for chronic low back pain. Guidelines published by several government and national organizations have recommended nonpharmacological and nonopioid pharmacological therapies for low back pain.

**Objective:**

The Pain eHealth Platform (PEP) pilot trial aims to test the feasibility of a highly innovative intervention that (1) uses an electronic health record (EHR) query to systematically identify a phenotype of obese, older adults with chronic low back pain who may benefit from Web-based behavioral treatments; (2) delivers highly tailored messages to eligible older adults with chronic low back pain via the patient portal; (3) links affected patients to a Web app that provides education on the efficacy of evidence-based, nonpharmacological, behavioral pain treatments; and (4) directs patients to existing Web-based health treatment tools.

**Methods:**

Using a three-step modified Delphi method, an expert panel of primary care providers will define a low back pain phenotype for an EHR query. Using the defined low back pain phenotype, an EHR query will be created to identify patients who may benefit from the PEP. Up to 15 patients with low back pain will be interviewed to refine the tailored messaging, esthetics, and content of the patient-facing Web app within the PEP. Up to 10 primary care providers will be interviewed to better understand the facilitators and barriers to implementing the PEP, given their clinic workflow. We will assess the feasibility of the PEP in a single-arm pragmatic pilot study in which secure patient portal invitations containing a hyperlink to the PEP Web app are sent to 1000 patients. The primary outcome of the study is usability as measured by the System Usability Scale.

**Results:**

Qualitative interviews with primary care providers were completed in April 2019. Qualitative interviews with patients will begin in December 2019.

**Conclusions:**

The PEP will leverage informatics and the patient portal to deliver evidence-based nonpharmacological treatment information to adults with chronic low back pain. Results from this study may help inform the development of Web-based health platforms for other pain and chronic health conditions.

**International Registered Report Identifier (IRRID):**

DERR1-10.2196/14525

## Introduction

Low back pain is the leading cause of disability worldwide and its prevalence increases with age [1]. The most commonly prescribed drug class for low back pain is opioids [2]. Prescription opioid use has numerous drawbacks with regard to the quality of life and has become a threat to public health. An estimated 17,029 people (5.2 per 100,000 people) died from prescription opioid overdoses in 2017 [3]. This is a particularly pressing concern for older adults due to the increasing incidence of prescription opioid misuse among members of this group [4,5]. Obese, older adults are significantly more likely to have chronic low back pain compared to their normal weight counterparts [6]. Obesity contributes to a higher frequency of intractable pain episodes [7], the use of stronger opioid medications compared to normal-weight adults with chronic pain [7], and increased pain severity [8].

Primary care providers (PCPs) are typically the first clinicians to see patients with pain and treat approximately 52% of patients with chronic pain [9]. Chronic pain is recognized as pain that persists past the normal healing time of 3 months [10]. Despite limited evidence to support their use, opioids are still prescribed in over 30% of primary care visits for chronic pain [11]. The American College of Physicians and the Centers for Disease Control has released guidelines for PCPs, which recommend nonpharmacological and nonopioid pharmacological therapies as the preferred initial treatments for chronic, noncancer pain [12,13]. However, older adults undergoing analgesic drug treatments for pain are more susceptible to the side effects caused by multiple comorbidities, age-related physiological changes, and altered pharmacokinetics and pharmacodynamics of analgesic drugs [14]. With recent efforts to increase legislation limiting opioid medications in many states [15,16], PCPs urgently need effective, evidence-based, nonpharmacological approaches for the management of chronic low back pain in adults.

There is a growing body of evidence showing that nonpharmacological behavioral treatment programs, which promote self-management techniques (like mindfulness meditation [17] and increasing physical activity [18]), are effective treatments for chronic pain. However, the traditional delivery of behavioral treatments relies on time-intensive, in-person sessions with a trained expert. This is a care model associated with numerous access-to-care barriers including time constraints, transportation, and cost [19].

Healthcare systems are tasked with the challenge of how to best implement evolving evidence-based guidelines for treating chronic health conditions like low back pain. The rapidly increasing use of the internet by older patients [20] and the growing presence of patient portals offer expanding opportunities to deliver Web-based behavioral treatments for pain [21]. Web-based portals allow patients to access their personal health information such as laboratory results, upcoming appointments, and medication information. Additionally, patients can use portals for bidirectional communication with their PCPs about their health status and treatments. To receive the maximum payment from Medicare, the Centers for Medicare & Medicaid Services electronic health record (EHR) Incentive Programs apply “meaningful use” criteria that requires PCPs to demonstrate that at least 5% of their patients use Web-based portals, strongly incentivizing healthcare systems to adopt and implement this technology [22,23]. A total of 48% of patients (n=50,190) between the ages of 50-79, seen at least once by a Wake Forest Baptist Health (WFBH) PCP during the 2018 calendar year, have a patient portal account.

Older adult-specific, Web-based treatment tools are quickly becoming commonplace [24-27]. Web-based behavioral treatments for pain have demonstrated efficacy [21] and are well positioned to address treatment barriers such as transportation, cost, time constraints, knowledge of existing Web-based health resources, and both patient and provider access to these resources. To this end, we are developing an innovative Pain eHealth Platform (PEP) that leverages clinical informatics and a patient portal to deliver evidence-based, nonpharmacological treatment information that builds patient knowledge of various behavioral pain treatments and directs patients to existing Web-based health treatment tools.

## Methods

### Overview

The form and function of the PEP will be developed in a systematic, patient-centered fashion. The conceptual model for the development of PEP and a description of the three-part PEP study is presented below. The study protocol was reviewed and approved by the Wake Forest School of Medicine Institutional Review Board (IRB00049293) in April 2018.

### Conceptual Model

**Figure 1:**
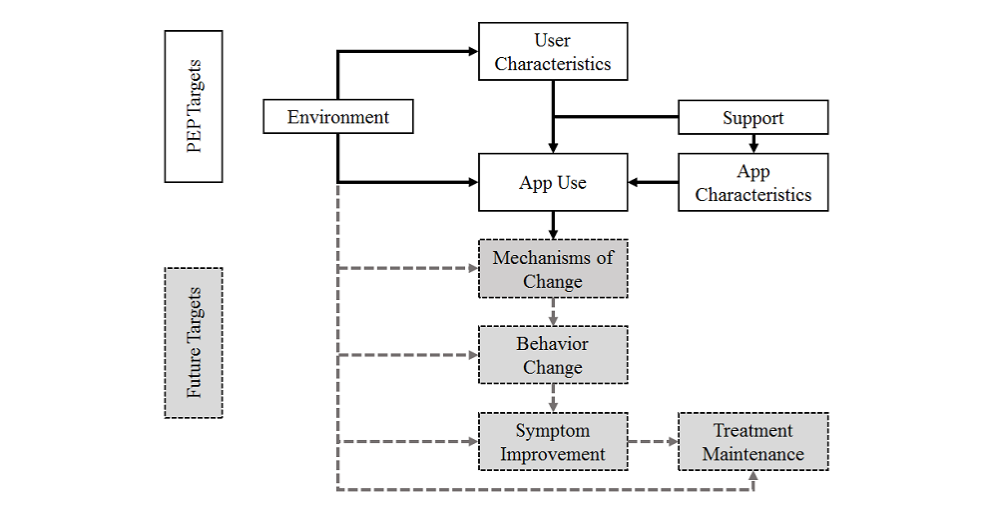
Behavior change model for Web-based interventions (adapted with permission from Ritterband et al [29]). PEP: Pain eHealth Platform.

The study is guided by social cognitive theory [28] and the Ritterband model [29] for digital interventions (Figure 1). As noted by Bandura [30], the initiation of successful behavior change is contingent on both the necessary skills and resources to engage in the behavior and confidence in one’s capability to successfully engage in the behavior, ie, self-efficacy [30]. The Ritterband model provides a useful framework for building knowledge and self-efficacy through the use of Web-based health platforms. In the model, *Application Use* is of central importance, as it is the strongest predictor of successful behavior change in internet-based treatments [31]. *Mechanisms of Change* such as improved knowledge and the development of self-efficacy, supported by the appropriate resources for behavior change, is anticipated to impact actual short-term behavior. Behavior maintenance is more likely when the behavior change promotes noticeable symptom improvement [32].

### Core Components of the Pain eHealth Platform

**Figure 2:**
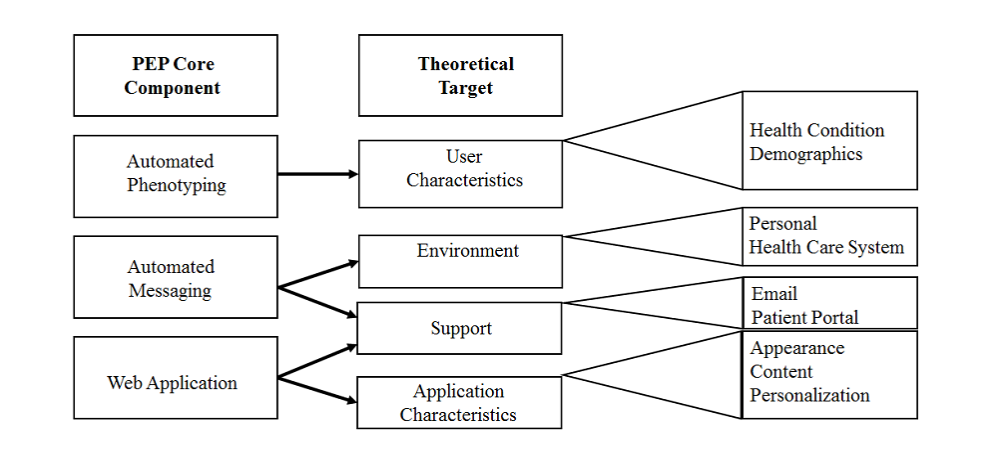
Pain eHealth Program core components and theoretical targets. PEP: Pain eHealth Platform.

The PEP will consist of an EHR query to identify patients who may benefit from the intervention, an invitation sent to patients via the patient portal to visit the PEP Web app, and the PEP Web app that includes three animated videos with evidence-based information about nonpharmacological treatments for low back pain including mindfulness meditation and physical activity (Figure 2).

### Electronic Health Record Query

To define the low back pain phenotype to be used for the EHR query, we will employ a three-step modified Delphi method. This method is an iterative process that uses a systematic progression of three rounds of voting and is effective for determining expert group consensus that does not necessitate multiple face-to-face meetings [33]. PCPs from the WFBH system, who spend at least 50% of their time seeing patients in the clinic and have been employed for ≥3 months will be recruited via email or telephone for the expert PCP panel. Content validation for the final EHR query can be satisfied by reaching 80% consensus from the expert panel at each step of the process [33].

Prior to initiating the modified Delphi process, the expert panel will be briefed about the benefits of mindfulness meditation and physical activities for chronic low back pain. In step 1 of the modified Delphi process, PCPs will be contacted via email to vote on a list of preliminary ICD-10 codes for low back pain. The list will also include patient characteristics such as age≥50-79 years, body mass index (BMI)≥30 kg/m^2^, and opioid use.

In step 2, the list of criteria that did not meet consensus from step 1 will be distributed to the group of PCP experts for consideration and another vote will be taken. Finally, in step 3, the expert consensus panel will convene via videoconferencing or face-to-face to deliberate and agree upon the final pain phenotyping for the EHR query. Provider participants will receive a US $50 incentive for their time and participation.

### Patient Portal Invitation

The portal invitation will be created using a user-centered design approach [34-36] to refine aspects of the invitation that will make it more effective in patient recruitment for the study. For example, we hypothesize that the sender of the portal invitation will be an important determining factor for viewing the embedded Web app content. Ten PCPs will participate in a 30-minute, semistructured interview to explore their thoughts regarding facilitators and barriers to implementing PEP (eg, time constraints, knowledge of existing Web-based health resources, and access to these resources). In addition, they will be asked for feedback regarding the wording, esthetics, and preferences of the patient portal invitation. They will be asked if they would prefer that the invitation come from “The WFBH Chronic Pain Study Team,” “The WFBH Primary Care Clinic, or “Name of Patient’s PCP” (Textbox 1). During the interview, we will explore why the PCPs prefer one option over another. We will invite PCPs who participate in the prior Delphi process to participate in the semistructured interviews, and the eligibility criteria will be the same. Provider participants will receive a US $25 incentive for their time and participation.

**Sample portal invitation.**

From: The WFBH chronic pain study team

Subject: New treatments for low back pain

Dear ____,

An automated analysis of your Wake Forest Baptist Health primary care record indicates you may benefit from new treatment options for low back pain. To learn more, *click here*.

### Pain eHealth PlatformWeb App Development

**Figure 3:**
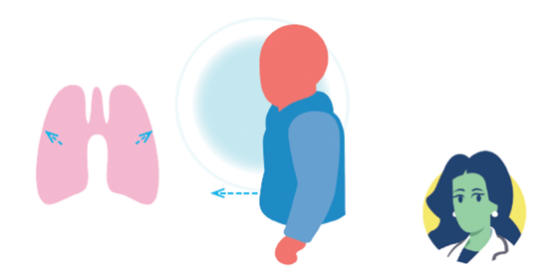
A screenshot from the mindfulness meditation video explaining the basic techniques of mindful breathing, as narrated by “Doc”.

The PEP Web app will be fully Web-based, requiring no downloading, and will be functionally available on both desktop and mobile browsers. Prior to beginning the PEP Web app program, patients will be asked to provide their highest level of education and to rate their low back pain on a sliding scale (0=no pain to 10=worst pain imaginable), on average, over the past week. The Web app will contain three animated videos narrated by a PCP named “Doc” who discusses non-pharmacological treatments for low back pain including mindfulness meditation [17,37] and physical activity [18] (Figure 3). These animated videos were originally created for the Mobile Intervention to Reduce Pain and Improve Health in Older Adults with Obesity (MORPH) study [34]. However, the content is broadly applicable to obese patients with a variety of chronic pain conditions. Upon viewing the animated videos, patients will be given links to additional Web-based mindfulness meditation resources [38,39] and 11 supplemental podcasts that provide tangible ways to increase physical activity created by our study team. These additional resources will help reinforce the concepts presented in the animated videos.

To develop the PEP Web app, we will conduct semistructured, qualitative interviews with obese, older adults with chronic low back pain. Because older adults are likely to have unique requirements, needs, and desires with regard to the Web app design and functionality within the PEP, a user-centered design process is essential [36]. At least 15 patients will sequentially participate in a Think-Aloud protocol: a 30-minute, individual, semistructured interview session in which they will be instructed to “act as though you are talking to yourself but loud enough for others to hear” [36,40]. Eligibility for patients include age≥50-79 years, BMI≥30 kg/m^2^, report of low back pain for ≥3 months, and English speaking. Excluded patients will have cognitive impairment, with a Montreal Cognitive Assessment [41] score<22. Eligible individuals will be asked to freely explore the Web app, with the aim of obtaining data related to app esthetics, functionality, and navigation. Audio recordings and detailed notes will be made during the semistructured interview, feedback will be discussed among team members, and modifications will be made to the Web app before the next participant completes the protocol. Audio recordings will be transcribed and coded, which will allow for tracking of changes in usability between participant interviews. Patient participants will receive a US $25 incentive for their time and participation.

### Pain eHealth Platform Implementation

**Figure 4:**
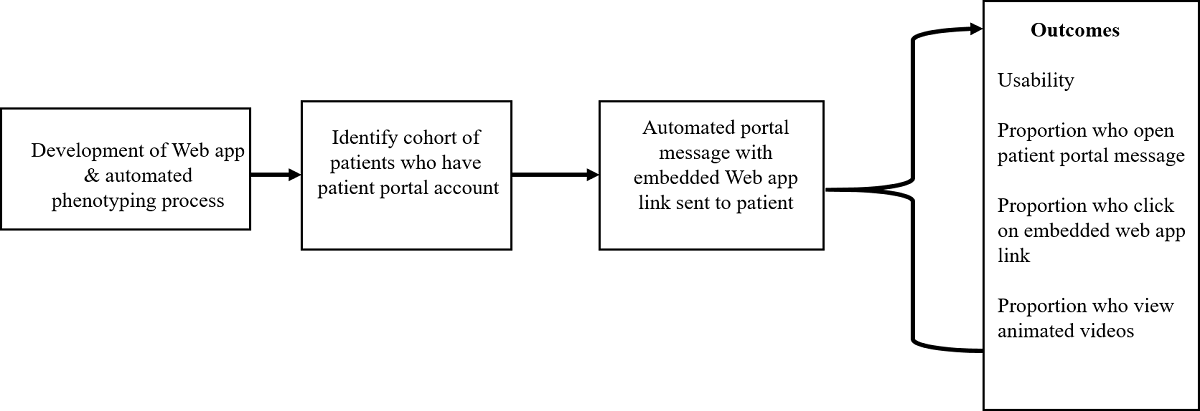
Overview of the Pain eHealth Program study design. EHR: electronic health record.

We will combine the EHR query, patient portal invitation, and PEP Web app into a seamless system to create the PEP (Figure 4).

Epic serves as the EHR for WFBH. The Web app will run external to Epic. The EHR query will be limited to data elements that are common to all EHRs. Variable names may differ between EHRs, but the query logic will be universal. The EHR query will be validated using iterative testing cycles of 20 randomly selected individuals (10 individuals who meet pain phenotype criteria and 10 individuals who do not meet pain phenotype criteria). Two reviewers who are blinded to the query output will independently review the EHR for these individuals. Disagreements between manual EHR review and query output will be reviewed, and the query will be modified, as needed. Testing cycles will continue until we reach 100% agreement between the query and manual chart review. The final EHR query will run weekly to identify potential candidates who already have an active patient portal account to receive a PEP portal invitation. Upon completion of the validation process, we will send a secure patient portal message to 1000 patients identified by the EHR query, inviting them to view three-animated videos and a list of recommended Web-based treatments about low back pain. The portal invitation will be sent two times, 1 week apart.

### Feasibility Outcomes

To measure usability of the Web app, participants will be prompted to complete items from the System Usability Scale (SUS) [42] after viewing the Web app content. The SUS provides a progressive set of options to assess the usability of a product or service. The SUS comprises 10 statements (eg, ease of use, confidence of use, and user-friendliness), each of which is scored 1-5 points on a Likert scale, from strongly agree to strongly disagree. Higher agreement scores indicate better usability, and scores from 70% to 100% represent good to best imaginable usability.

Secondary outcomes of using the PEP include feasibility and process measures including the reach of our portal outreach strategy (proportion of patients who read the portal invitation, proportion who visit the Web app link within 1 month of the invitation being sent, and proportion who click on all the Web-based treatment links). Effectiveness of the portal message invitation will be measured by the proportion of those who read the message that clicked on the Web app hyperlink. Lastly, a follow-up survey will be sent to patients 1 week after completion of the Web app, asking if they have tried any of the recommended techniques. Reponses to this survey will provide initial evidence regarding uptake of the recommended nonpharmacological treatments. All outcome data will be obtained by querying the EHR and Web app databases.

### Analysis Plan

Descriptive statistics for continuous variables (mean and SD) or categorical variables (frequencies) will be presented for patient characteristics and the outcome measures. Multiple linear regression will be used to examine potential associations between SUS scores and participant characteristics such as age, race, gender, and BMI. Logistic regression will be used to determine which participant characteristics are associated with the proportion who read the portal invitation, those who click on the embedded Web app PEP link, and those who click on all the Web-based included treatments links. These analyses will determine which patients are more receptive to the intervention and which patients may require alternative strategies or messages. A large population of 1000 patients will allow the estimate of primary and secondary outcomes and will provide sufficient variability to plan a future larger study. If 300 patients (30%) complete the SUS, it will be possible to estimate the average usability within 2.8%, with 95% confidence.

### Potential Limitations

Internet use among older adults is increasing, with 67% of those aged ≥65 years reporting internet use [20]. However, internet use in this population varies widely by socioeconomic status. Although almost half of patients between the ages of 50 and 79 years have an active patient portal at WFBH, we realize that there may be important demographic differences between portal users and nonusers. We acknowledge that adults aged 70-74 years are less likely to have an active patient portal account than those aged 65-69 years. In addition, non-Hispanic white individuals and Chinese seniors are less likely to have registered patient portal accounts than black, Latino, and Filipino seniors [43]. However, there is growing evidence that adults with multiple chronic health conditions are more likely to access electronic personal records, including patient portals, compared to adults with no chronic health conditions [44]. Moreover, older adults who use patient portals often use fewer features relative to younger populations. In this regard, the PEP Web app will be developed in a user-centered fashion to ensure ease of usability among the target population. Additionally, PCPs are under tremendous time and resource constraints in the clinic. Thus, exploring facilitators and barriers to the implementation of the PEP by PCPs (as described in the PEP Web App Development section) is a vital step of this proposed project.

## Results

Qualitative interviews with primary care providers were completed in April 2019. Qualitative interviews with patients will begin in December 2019.

## Discussion

This is the first study to examine the usability and reach of a patient portal outreach strategy for delivering evidence-based, nonpharmacological treatment information to adults with chronic low back pain. Given the limited efficacy and frequent adverse effects of opioid medications used to treat chronic low back pain, PCPs require methods for implementing evidence-based, nonpharmacological therapies in their clinical practices without substantial additional burden.

This study is not designed to determine the effectiveness of the Web app. However, the findings from the follow-up survey will provide initial evidence regarding uptake of recommended nonpharmacological interventions. The long-term goals of this project are to test the efficacy of the PEP in a large-scale, hybrid, efficacy-implementation trial that will measure clinical outcomes including pain, physical function, and opioid prescription use over time and to adapt this research platform to a variety of other chronic health conditions including other pain conditions, for which there are established evidenced-based treatment guidelines.

## Abbreviations

EHR: electronic health record
MORPH: Mobile Intervention to Reduce Pain and Improve Health in Older Adults with Obesity
PEP: Pain eHealth Platform
PCP: primary care providers
SUS: System Usability Scale
WFBH: Wake Forest Baptist Health
